# The subclonal complexity of *STIL-TAL1+* T-cell acute lymphoblastic leukaemia

**DOI:** 10.1038/s41375-018-0046-8

**Published:** 2018-03-20

**Authors:** Caroline L Furness, Marcela B Mansur, Victoria J Weston, Luca Ermini, Frederik W van Delft, Sarah Jenkinson, Rosemary Gale, Christine J Harrison, Maria S Pombo-de-Oliveira, Marta Sanchez-Martin, Adolfo A Ferrando, Pamela Kearns, Ian Titley, Anthony M Ford, Nicola E Potter, Mel Greaves

**Affiliations:** 1 Centre for Evolution and Cancer, The Institute of Cancer Research, London, UK; 2 Paediatric Haematology-Oncology Program, Research Centre, Instituto Nacional de Câncer, Rio de Janeiro, Brazil; 3 Institute of Cancer and Genomic Sciences, University of Birmingham, Birmingham, UK; 4 Wolfson Childhood Cancer Research Centre, Northern Institute for Cancer Research, Newcastle University, Newcastle-upon-Tyne, UK; 5 Department of Haematology, University College London Cancer Institute, University College London, London, UK; 6 Institute for Cancer Genetics, Columbia University, New York, NY 10032 USA

## Abstract

Single-cell genetics were used to interrogate clonal complexity and the sequence of mutational events in *STIL-TAL1*+ T-ALL. Single-cell multicolour FISH was used to demonstrate that the earliest detectable leukaemia subclone contained the *STIL-TAL1* fusion and copy number loss of 9p21.3 (*CDKN2A/CDKN2B* locus), with other copy number alterations including loss of *PTEN* occurring as secondary subclonal events. In three cases, multiplex qPCR and phylogenetic analysis were used to produce branching evolutionary trees recapitulating the snapshot history of T-ALL evolution in this leukaemia subtype, which confirmed that mutations in key T-ALL drivers, including *NOTCH1* and *PTEN*, were subclonal and reiterative in distinct subclones. Xenografting confirmed that self-renewing or propagating cells were genetically diverse. These data suggest that the *STIL-TAL1* fusion is a likely founder or truncal event. Therapies targeting the *TAL1* auto-regulatory complex are worthy of further investigation in T-ALL.

## Introduction

Single-cell genetics in haematopoietic [1–3] and other cancers [4, 5] have revealed substantial intraclonal complexity. In general, this diversity reflects evolutionary phylogenies with derivative subclones branching off from founder precursors [6]. Architectural population diversity in cancer has important implications for reservoirs of cells involved in progression of disease and drug resistance therapy. Bioinformatic derivations of evolutionary trees can reveal the most likely sequence of genetic events and distinguish mutations that are present in all cancer cells, as truncal or founder events, versus those that are secondary and subclonally distributed [7–9]. This in turn carries implications for minimal residual disease (MRD) monitoring and targeted therapy.

Few such studies have been performed to date in T-ALL, although comparative genetic profiling of diagnostic, xenograft and relapse samples confirms clonal complexity [10, 11]. T-ALL is biologically diverse reflecting levels of differentiation arrest within the thymus and distinctive genetic lesions [12]. We elected to study a single, common subtype of T-ALL, namely those with *STIL-TAL1* fusion. We used multicolour FISH and single-cell multiplex quantitative-PCR (qPCR) to determine the phylogenetic architecture of diagnostic samples and to infer the order of genetic events comparing *STIL-TAL1* fusion, which we postulated as a founder lesion, with other common genetic lesions including *CDKN2A* loss, *PTEN* mutation or loss and *NOTCH1* mutation. In selected cases, we compared the clonal architecture of xenotransplanted samples with that observed in the diagnostic sample. This enabled us to infer the subclonal origins and genetic diversity of cells with propagating or stem cell activity.

## Materials and methods

### Patient samples

Diagnostic DNA of 19 T-ALL cases aged 1–24 years and 1 cell line (RPMI 8402) known to have the *STIL-TAL1* rearrangement were available. The study was conducted in accordance with the Declaration of Helsinki and appropriate consent and ethical approval for the study was obtained (Ethics approval numbers CCR2285 and 16/SE/0219).

### T-ALL molecular screening and cloning

Diagnostic DNA from all *STIL-TAL1* cases was analysed for mutations in known T-ALL mutational hotspots in *NOTCH1* (exons 26, 27 and 34), *FBXW7* (exons 9 and 10), *PTEN* (exon 7) and *IL7R* (exon 6) using previously published methods [13–16]. All diagnostic samples were analysed by SNP-array to identify genomic losses and gains using the Affymetrix SNP 6.0 platform. Genotyping and generation of QC data were performed in Genotyping Console^TM^ v4.1.4 software (Affymetrix). CNAG version 3.3.0.1 beta was used to normalise output to a self-reference (patient remission DNA) or via a batch pairwise analysis using sex-matched control samples. The *STIL-TAL1* patient-specific gene fusion was sequenced for the three cases that underwent single-cell genotyping analyses using previously published methods [17]. The TA Cloning Kit^®^ (Invitrogen by Life Technologies^TM^) was used for cloning experiments according to the manufacturer’s instructions.

### Next-generation sequencing (NGS)

Whole exome sequencing (WES) was undertaken by Oxford Gene Technology. See Supplementary Methods for details and bioinformatics. Any genomic ‘drivers’ included in the single-cell genotyping experiments were validated with Sanger sequencing using custom primers designed using Primer Blast (Table S1).

### Fluorescence in situ hybridisation

Fixed cytospins were prepared from archived viable cells and interphase FISH was performed with patient-specific FISH probes for the various copy number losses using in-house FISH probes (Table S2) and previously described methods [18]. See Supplementary Methods for details.

### Bioinformatic assessment of RAG recombinase activity at *PTEN* indel breakpoints

MEME Suite 4.11.4 [19] was used to conduct an agnostic search for the RAG recombinase consensus heptamer and nonamer and also for the tetramer sequence (CACA) identified [20] as being recurrently present at RAG-mediated breakpoint sequences. We also used a weighted matrix algorithm (code availability—script kindly provided by the laboratory of Dr Papaemmanuil) to generate RAG recombination signal sequences (RSS) scores for each deletion breakpoint of interest. Essentially, this ascribed a likelihood score or weight to each base pair in the putative heptamer-spacer-nonamer sequence of interest according to the likelihood of deviation from consensus based on what the base pair is for the heptamer/nonamer and the number of bases rather than base choice per se for the spacer (score details outlined in ref. [21]).

### Single-cell genotyping and single-cell Sanger sequencing

Single-cell genetic analysis was performed using stored viable cells for cases CUL76, 6116 and 6030 and paired xenograft material. Our previously established multiplex qPCR approach was used [2] with minor modifications. See Supplementary Methods for details.

### Xenograft material

Limited archived xenograft DNA and single-cell material prepared from xenograft bone marrow was available on samples CUL76, 6030 and 6116. Material generated using NOG (NOG-ShiSCID-IL2gamma null) mice was available for samples 6030 and 6116 and using NRG (NOD-*Rag1*^*null*^*IL2rg*^*null*^, NOD rag gamma) mice for sample CUL76. In all cases, 1 × 10^6^ cells were injected and experiments were performed on material stored from primary passage bone marrow.

## Results

### Molecular screening on *STIL-TAL1+* T-ALL samples for recurrent drivers

Nineteen *STIL-TAL1+* cases and one cell line were screened for common genetic rearrangements using copy number profiling and Sanger sequencing. Data on Brazilian samples BR75, BR74, S1 and S2 have been previously published [22, 23]. Four cases also underwent WES to identify both known driver targets for inclusion in single-cell experiments and novel drivers. Results of molecular screening are summarised in Table 1 (further details available from Tables S8, S9 and S10). Mean target coverage across the samples (and paired remission samples) that underwent WES was 95×–126× (average 109×). Total of 7–10 protein altering SNVs and 3–5 protein altering indels (detectable at a read depth >20) were detected per sample (Tables S11 and S12). All drivers incorporated in to single-cell experiments were validated with qPCR or Sanger sequencing.

**Table 1:** The genomic landscape of *STIL-TAL1* T-ALL

	

Recurrent drivers in *STIL-TAL1* T-ALL based on Sanger sequencing and copy number data in 20 *STIL-TAL1* T-ALL samples. Blue: copy number loss; red: copy number gain; orange: mutation; white: wild type. Details of putative drivers identified by WES are also shown for samples 6030, 6116, CF5 and CF10. Full details of all alterations identified by WES are included in Supplementary Data as well as localisation of all mutations noted in Table 1 (Supplementary Tables S9–S12)

In keeping with previous work highlighting that cases from the *TAL/LMO* gene expression subgroup tend to have a higher incidence of *PTEN* mutations and a lower incidence of *NOTCH1* mutations [24], within our cohort of 20 patients (combining *PTEN* inactivation due to exon 7 mutation or copy number loss) we detected a frequency of *PTEN* inactivation of at least 40% (around double the frequency of *PTEN* inactivation (22%) detected in a recent study of 145 T-ALL cases using heteroduplex analysis, mutations and SNP arrays) [25]. Additionally in the cases that underwent WES, mutations in *PTEN* outside the hotspot of exon 7 were detected (exon 8 in sample 6030 and exon 5 in CF10), suggesting that estimates based on copy number analysis and *PTEN* exon 7 sequencing may be an underestimate. A high frequency of copy number losses of 9p (95%, incorporating *CDKN2A/CDKN2B* locus) and 6q (30%) was also observed and these were identified as key drivers to include in single-cell genotyping studies.

Exome sequencing of four cases defined relevant known and potential T-ALL drivers to include in single-cell analysis experiments. Novel T-ALL potential drivers were detected: mutations in *BMPR1A, FREM2* and *PIK3CD*. *BMPR1A* (bone morphogenetic protein receptor, type 1A) is a polyposis associated gene—a type 1A transmembrane serine/threonine kinase listed in the cosmic cancer gene census of genes functionally linked to cancer and *PIK3CD* is a subunit of phosphatidylinositol-4,5-bisphosphate 3-kinase a key member of the *PTEN*-PI3 kinase pathway. Both 6030 and 6116 contained one mutation per sample in *FREM2* and one of these mutations (found re-iteratively in sample 6116) is found in the cosmic database as a somatic mutation in a GI carcinoma (COSM287123). Additionally, this gene was recurrently but non-significantly mutated in three T-ALL samples in a large exome T-ALL sequencing study [26]. The gene encodes an integral membrane protein containing many chondroitin sulphate proteoglycan element repeats and calx-beta domains (although the identified mutations lie outside these domains, Figure S3). In view of the possible association with T-ALL, and the emerging role of the micro-environment/extracellular matrix in leukaemia and cancer, we included *FREM2* mutations in our single-cell studies.

### Reiterative inactivation of *PTEN*

**Fig. 1:**
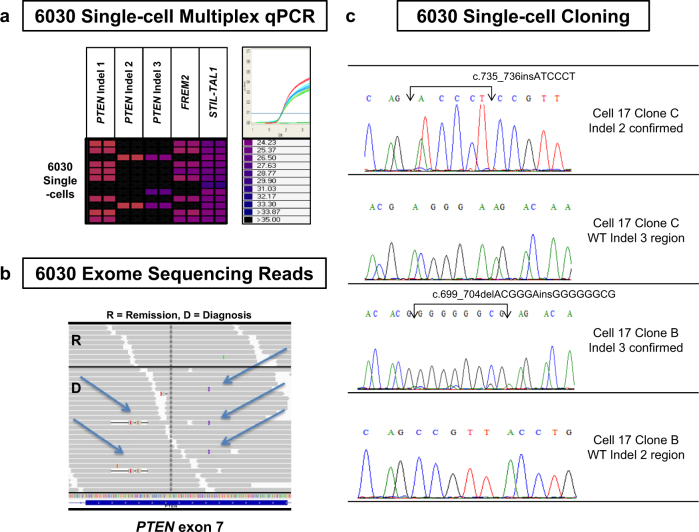
Reiterative bi-allelic deletion of *PTEN*. **a** Multiplex qPCR data show that *PTEN* indels 2 and 3 are localised within the same single cells. The coloured boxes at the junction between the vertical qPCR assays (run in duplicates) and the horizontal individual cells represent mutation-specific amplification with the colour of each box correlating with the level of amplification (raw Ct value). Black represents no amplification. **b** Reiterative *PTEN* exon 7 indel mutations are seen within the raw data reads of whole exome sequencing data for sample 6030. **c** Cloning of *PTEN* exon 7 from representative single cells demonstrated that these indels are present on separate alleles as one single-cell clone contains indel 2 but is wild-type (WT) for indel 3, whereas another single-cell clone contains indel 3 but is WT for indel 2 demonstrating bi-allelic inactivation of *PTEN*. Cell number 17 refers to experimental single-cell DNA plate position

Sanger sequencing and NGS data for samples 6030 and CF5 suggested the presence of multiple low-level *PTEN* exon 7 indels running in parallel (Fig. 1a, b). Parallel work by a collaborating group using HPLC wave technology (heteroduplex analysis) [25] also led to similar conclusions. Cloning experiments validated at least four independent indels for sample 6030. Sample CF10 also had two *PTEN* indels in exons 5 and 7 (detected by WES and Sanger sequencing). The multiple *PTEN* indels resulted functionally in the generation of stop codons in all but one of the mutations analysed (10/11; Table S13). Given the striking reiterative inactivation of *PTEN*, we hypothesised that these small structural alterations could be RAG-mediated given the involvement of aberrant RAG in the formation of the *STIL-TAL1* fusion and *CDKN2A* deletions and the observation that RSS elsewhere in *PTEN* have been implicated in the formation of small microdeletions [27]. However, we did not find bioinformatic evidence to support this explanation with maximum RSS scores of 6.75 (Table S14) compared to >8.55 in B cell precursor (BCP)-ALL samples using the same weighted matrix algorithm [20]. Only one sample (CF10) had the RAG-associated CACA tetramer identified in Papaemmanuil et al. (2014) close to the mutation breakpoint.

### Single-cell studies in *STIL-TAL1+* T-ALL

#### Multicolour FISH

**Fig. 2:**
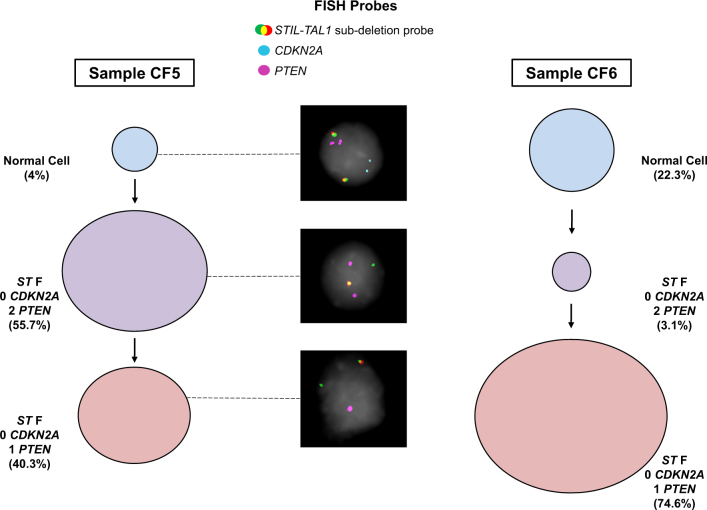
Multicolour FISH in *STIL-TAL1* T-ALL. Examples for evolutionary trees for samples CF5 and CF6 are shown. Percentages represent the frequency of each subclone as assessed by single-cell multicolour FISH with circle size representing relative frequencies. Photos shown are from sample CF5 using the following FISH probes: *STIL-TAL1* (labelled spectrum red-spectrum green); p16.1 as a marker of *CDKN2A* loss (labelled Cy3, coloured blue in diagram); *PTEN* (labelled Biotin-Cy5, coloured pink in diagram). Note the *STIL-TAL1* FISH probe is a subdeletion probe. Normal cells have two co-localised red-green signals. Cells with the *STIL-TAL1* fusion due to 1p33 deletion lose one red signal while retaining the control green signal. ST F: *STIL-TAL1* Fusion. Numbers (0, 1, 2) next to gene name (*PTEN* or *CDKN2A*) represent copy number results by FISH, e.g., 2 *PTEN*, two copies of *PTEN*, 0 *CDKN2A*, zero copies of *CDKN2A*

We investigated the order of acquisition of CNAs with reference to the *STIL-TAL1* fusion in samples 6030, CF5, CF6 and HK328. In the majority of cases, the earliest ancestor subclone contained the *STIL-TAL1* fusion in combination with bi-allelic loss of 9p21.3 (containing *CDKN2A* locus) as shown for samples CF5 and CF6 (Fig. 2). In contrast with BCP-ALL [9] in the majority of cases 9p21.3 could not be separated in time from the presumed founder gene fusion/translocation. Additional copy number losses including 6q and *PTEN* occurred in a secondary and subclonal fashion. However, in sample 6030 the earliest detectable subclone contained *STIL-TAL1+* cells with 1 copy of 9p21.3. This observation was validated using two independent FISH probes and the clone was also detected in xenograft bone marrow derived from sample 6030 (Figure S1a).

#### Single-cell multiplex qPCR

Although multicolour FISH provides proof-of-principle evidence for clonal heterogeneity in T-ALL, the number of driver genetic events that can be examined is limited and mutations in key T-ALL signalling pathways cannot be incorporated. We undertook a detailed single-cell genotyping study on three samples that represented all the key *STIL-TAL1* genetic driver events identified in this study and which had archived paired xenograft material available. Archived diagnostic leukaemia cells from cases 6030, 6116 and CUL76 underwent high-throughput single-cell multiplex qPCR analysis allowing simultaneous investigation of the patient-specific *STIL-TAL1* gene fusion in each case along with indels, SNVs and CNA designated driver status for each leukaemia sample. Quality control assessments and analysis were performed as previously described [2].

**Fig. 3:**
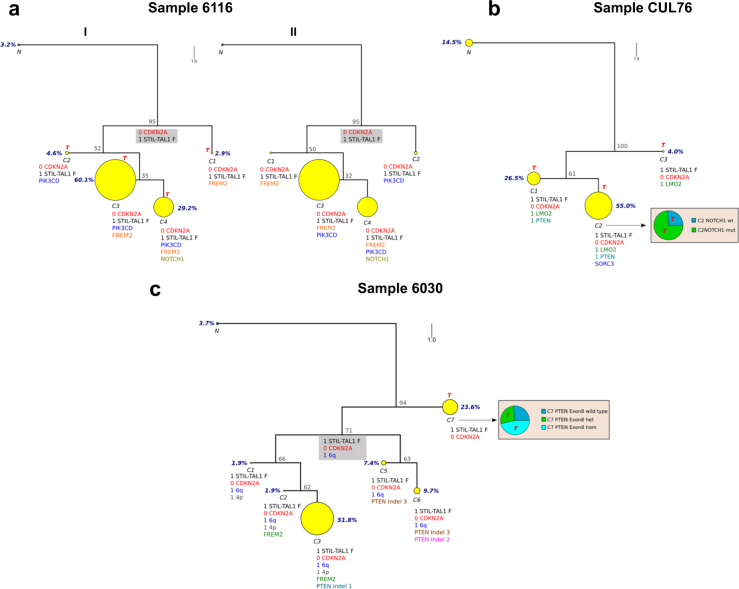
**a**–**c** Single-cell multiplex qPCR evolutionary analysis samples 6030, 6116 and CUL76. Evolutionary trees generated by bioinformatics analysis of single-cell data are shown. Gene names of drivers tracked are shown next to the subclones. Presence of a gene name indicates gene mutation present in the case of SNVs or indels. Where the driver is a deletion number of copies of gene present (0 or 1) is shown. Yellow circles represent leukaemic subclones and a black circle and the N label indicates the normal state. The size of the circle is proportional to the number of cells in each subclone and the detected genetic markers are listed below each circle. Red T = subclones that read-out in xenotransplants (detailed xenograft single-cell data in Supplementary Table 16). Grey boxes represent inferred subclones; these are groups of cells, which have died out, been outcompeted or if still present, exist at low frequencies below the level of reliable detection using this approach. Tree branch lengths are directly proportional to the number of evolutionary changes inferred and the points at which the branches diverge (nodes) represent the ancestor state of a clonal clade; a monophyletic group, which includes all descendants of the ancestor. The number in grey colour at each node indicates the bootstrap value. The phylogeny shows how the clonal expansion has evolved from a common ancestor toward the observed states. Note that for case CUL76 as a limited number of drivers were tracked based on mutation screening and copy number data another known mutation present in this sample (*SORC3*, passenger mutation) was included to aid assessment of clonal structure. **a** Case 6116 (*N* = 308 cells). The root of the tree harbours the *STIL-TAL1* fusion (F) and a homozygous *CDKN2A* deletion. Two equally parsimonious trees (I and II) are generated which differ as to whether the *FREM2* or *PIK3CD* mutated clone gives rise to the latest detectable C4 clone. The *NOTCH1* mutation is a subclonal event. **b** CUL76 (*N* = 151 cells). The root of the tree harbours the *STIL-TAL1* F and a homozygous *CDKN2A* deletion and a *LMO2* deletion in this case. *PTEN* inactivation, through copy number loss, and *NOTCH1* mutation are subclonal. The *NOTCH1* TAD domain exon 34 mutation was only detected in clone C2 (*NOTCH1* mutation status determined by single-cell Sanger sequencing of 44 single cells). The pie chart shows the proportions of C2 subclone cells that are either wild-type (WT) or *NOTCH1* mutated. **c** 6030 (*N* = 216 cells). The root of the tree harbours the *STIL-TAL1* F and a homozygous *CDKN2A* deletion. *PTEN* mutations are subclonal and re-iterative (3 *PTEN* indels in exon 7—labelled *PTEN* indels 1, 2 and 3 plus one *PTEN* indel in exon 8). The C6 subclone contains two independent *PTEN* exon 7 mutations. *PTEN* exon 8 mutation was only detected in clone C7 (*PTEN* exon 8 mutation status determined by single-cell Sanger sequencing of 37 single cells). The pie chart shows the proportions of C7 subclone cells that are WT, heterozygous *PTEN* exon mutated or homozygous *PTEN* exon 8 mutated

Phylogenetic trees for these three diagnostic cases constructed from single-cell data using the principle of maximum parsimony are shown in Fig. 3a–c. In all cases the root of the tree contains a common ancestor cell containing the patient-specific *STIL-TAL1* fusion and copy number loss of *CDKN2A*. Other key drivers are subclonal but in some cases re-iterative in keeping with observations made in prior studies of B cell leukaemias and solid tumours [2, 9, 28–30]. The *STIL-TAL1* assays used were specific for the patient-specific fusions (Table S15) and tested against both normal DNA and other *STIL-TAL1* positive material to confirm assay specificity. This study provides the first evidence that a single *STIL-TAL1* fusion occurs per case (previous studies assessing stability through xenograft passage have used chromosome 1p copy number as a surrogate for the gene fusion but this would not differentiate the presence of multiple overlapping fusions with relatively conserved mutation breakpoint region). *NOTCH1* mutation and *PTEN* mutation are secondary and subclonal events in the case studies. The multiple *PTEN* mutations noted in sample 6030 were tracked at single-cell level and single-cell cloning and Sanger sequencing were used to demonstrate unequivocally that in clones with >1 mutation, mutations occurred on independent alleles suggesting a selective pressure for bi-allelic inactivation of *PTEN* (Fig. 1c).

The use of xenograft models can be used to assess clonal heterogeneity and evolution in the leukaemia initiating or so called ‘cancer stem-cell’ compartment providing insight in to genetic driver stability and evolutionary pressures on clonal selection. Single-cell assessment of xenograft bone marrow in the three cases examined demonstrated contrasting models of clonal evolution, although it is acknowledged that limited conclusions can be drawn from the single xenograft per case experiments performed. Clones that read-out in the xenograft are designated ‘T’ status in the evolutionary tree diagrams (Fig. 3a–c). In cases 6116 and CUL76, multiple clones read out in the xenograft confirming the position of *NOTCH1* as a subclonal driver and demonstrating multiple competing subclones in the leukaemia initiating cell compartment. However, in case 6030 a single dominant clone (C7) predominated containing the *STIL-TAL1* fusion, bi-allelic 9p21.3 deletion and a *PTEN* exon 8 mutation, which was present in both heterozygous and homozygous form. The proportion of cells within this subclone with a homozygous (as opposed to heterozygous) *PTEN* exon 8 mutation was higher in the xenograft than the diagnostic sample (88% versus 47%) and all xenograft single cells had either the heterozygous or homozygous mutation (i.e., no *PTEN* exon 8 wild-type cells detected based on Sanger sequencing of 78 single cells). The clones with *PTEN* exon 7 mutations were not detected in the xenograft. This validated bulk DNA sequencing data from the xenograft material which demonstrated the homozygous *PTEN* exon 8 mutation and wild-type *PTEN* exon 7 analysis (data not shown).

Comparison of 6030 xenograft single-cell FISH and multiplex qPCR data initially appeared to generate conflicting results with FISH data suggesting the presence of a clone with 1 copy of 9p21.3 in both diagnostic and xenograft material but single-cell data demonstrating bi-allelic loss in all subclones. Examination of SNP copy number data resolved this discrepancy, which was due to one of the 9p21.3 deletions being smaller than the size of the FISH probe so that the FISH subclone containing ‘1 copy’ of 9p21.3, 2 copies of 4p/6q (Figure S1a) actually corresponded to clone C7 in multiplex qPCR data (Fig. 3c), i.e., *CDKN2A* actually demonstrated bi-allelic deletion when small qPCR assays were used to assess copy number status. Examination of paired diagnosis/xenograft copy number data for case 6030 also demonstrated that *CDKN2A* loss is a subclonal event as with regard to the loss of the second allele of *CDKN2A* at least two independent clones had emerged with distinct breakpoints (Figure S1b). Since multiplex qPCR data for *CDKN2A* was based on copy number assays, it is possible that multiple *CDKN2A* deletions may exist in any one sample, which would not be detected using the analysis method used.

## Discussion

These single-cell genetic analyses allow us to infer phylogenetic trees describing clonal evolution. Technical limitations mean that interpretation of these data carries the caveat that we will have under-estimated clonal complexity. Our genetic markers are also limited. More genetically distinct subclones will exist than we currently detect. For case 6116 due to the limited genetic markers used we were unable to determine whether the re-iterative mutation event was *PIK3CD* or *FREM2* mutation but did validate the bioinformatic analysis with single-cell Sanger sequencing analysis of *FREM2* and *PIK3CD* mutation in single cells of the diagnostic and xenograft samples to confirm that re-iterative mutation had occurred (Figure S2).

Despite these caveats, several informative conclusions can be drawn. A consistent feature is that *STIL-TAL1* fusion and *CDKN2A* loss are both early or truncal events, in contrast to other recurrent genetic changes including *NOTCH1* and *PTEN* mutation that are secondary and subclonal. These observations have implications for selection of mutations for minimal residual disease tracking and as targets for therapy. Given the position of the *STIL-TAL1* fusion in leukaemia evolution, therapies targeting the *TAL1* regulatory complex [31] are worthy of further investigation.

It is difficult to discern from our study whether *STIL-TAL1* or *CDKN2A* loss is an initiating event, or which comes first. The most ancestral cell in the phylogenetic structure has *STIL-TAL1* fusion plus loss of both *CDKN2A* alleles. However, the latter are distinctive and presumed independent events, but only one clone-specific *STIL-TAL1* fusion exists. This finding suggests that at least one of the *CDKN2A* allele deletions occurs subsequent to *STIL-TAL1* fusion. This is in keeping with previously published work demonstrating reiterative *CDKN2A* deletions in *STIL-TAL1* cases when breakpoints of 9p21.3 deletions in paired diagnostic/xenograft/relapse material were examined [11]. We note that both *STIL-TAL1* fusion and *CDKN2A* loss are likely to involve ‘off target’ RAG-dependent mutational mechanisms.

The clonal architectures in *STIL-TAL1*+ ALL cases (Fig. 3) for which we had genome sequencing data showed branching structure as previously described in B cell precursor ALL [9]. To some extent, this is driven by reiterative mutations of the same driver genes and resultant parallel clonal evolution. This is evident with several driver genes but most clearly with *PTEN* in patient 6030 (Fig. 3c). Cloning these multiple mutations from single cells confirmed their uniqueness (Fig. 1c). In BCP-ALL, reiterative copy number changes (e.g., in *ETV6, PAX5, CDKN2A* or *BTG1*) are the consequence of RAG-mediated mutation, followed by selection [9, 20, 30]. We found no bioinformatics support for RAG involvement in the *PTEN* mutations we identified. We note, however, that RSS for RAGs have been previously implicated in *PTEN* small, microdeletions [27]. Irrespective of the mutational mechanisms involved in these *PTEN* mutations, we conclude that in *STIL-TAL1* ALL there is a strong selective pressure for these genetic lesions, most likely related to epistasis or a strong functional complementarity between *PTEN* inactivation/loss, *STIL-TAL1* fusion and *CDKN2A* loss.

The objective, in our limited xenograft studies, was to determine the subclonal origin and genetic diversity of propagating or self-renewing cells in *STIL-TAL1*+ ALL. The data indicate that multiple subclones read out in the mice reflecting, we suggest, the existence of genetically diverse stem cells. In patient 6116 (Fig. 3a), all four diagnostic subclones read out in the transplants and in patient CUL76 (Fig. 3b) all three subclones. Patient 6030 gave a different result however. One dominant subclone (subclone C7; Fig. 3c) read-out of the seven that existed in the diagnostic sample. However, the diagnostic subclone C7 was heterogeneous with respect to *PTEN* exon 8 status and this directly was also reflected in the transplant read-outs, indicating that two small subclones of clone C7 had propagated in the mice, providing further evidence for selection favouring *PTEN*-mutated clones. Genetically diverse propagating or stem cells were similarly demonstrated in B cell precursor ALL [9, 32] and in glioblastoma [33] and are likely to be a common feature in cancer [34, 35]. The implication of this observation is that multiple subclones harbour the proliferative potential to fuel progression of disease, relapse and drug resistance.

## Electronic supplementary material

Supplementary Data CLEAN(DOCX 83 kb)

Figure S1(PPTX 691 kb)

Figure S2a,b(PPTX 154 kb)

Figure S3(PPTX 92 kb)
