# Supplementary material for: The subclonal complexity of STIL-TAL1+ T-cell acute lymphoblastic leukaemia
Source: Leukemia. 2018 Mar 20;32(9):1984–93. doi: 10.1038/s41375-018-0046-8 (PMC6127084; doi:10.1038/s41375-018-0046-8)
Supplement: Supplementary file 1 — Supplementary Data CLEAN(DOCX 83 kb) [file 41375_2018_46_MOESM1_ESM.docx]

**Supplementary Information**

**MATERIALS AND METHODS**

***Next generation sequencing (NGS)***

In brief, capture was performed using the Agilent SureSelect Human All Exon V5 kit following manufacturer’s procedures (Agilent) and sequenced with Illumina paired-end sequencing (protocol v1.2) run on an Illumina HiSeq 2000, according to manufacturer’s protocols. Exome analysis was completed in OGT’s exome pipeline. Reads were aligned to the human genome reference sequence GRCh37 using bwa 0.6.2.^1^ Somatic SNP and somatic indels (insertion and/or deletion) were identified between remission and diagnostic samples using VarScan2. Local realignment was performed around indels with the Genome Analysis Toolkit (GATK v1.6) IndelRealigner.^2^ Indels and SNPs were hard-filtered according to Broad Institute best-practice guidelines to eliminate false positive calls and variant annotation was performed with a modified version of Ensembl Variant Effect Predictor.^3^ We examined somatic mutations predicted to be protein altering by visual inspection in Integrative Genomics Viewer (IGV, The Broad Institute) to exclude any SNPs not filtered out by bioinformatics analysis.

***Fluorescence in situ hybridisation***

Prior to use, localisation of probes to relevant cytobands was confirmed by hybridisation to normal metaphase spreads, and Hybridisation to normal and control cell line interphase nuclei was used to determine FISH probe sensitivity and specificity. FISH probes were differentially labelled with spectrum red-dUTP, spectrum green-dUTP (both Enzo Life Sciences), Cy3-dUTP (Amersham^TM^ GE Healthcare, UK) or biotin-16-dUTP (Roche diagnostics) detected with Cy5-Streptavidin (Amersham^TM^ GE Healthcare, UK). Fluorescent signals were viewed using an Olympus BX61 microscope equipped with narrowband pass filters for DAPI, spectrum aqua, FITC/spectrum green, spectrum red/Cy 3.5, Cy3 and Cy5.

***Single-cell genotyping and single-cell Sanger sequencing***

In brief, fixed propidium iodide (PI) labelled single-cells were sorted in to individual wells of 96-well plates by fluorescently activated cell sorting (BDFACSAria I SORP). Eleven control cord blood cells were included in each plate. After cell lysis multiplex target specific DNA amplification was completed using custom primers (Table S3) or commercially available DNA copy number assays (Life Technologies, Table S4) to amplify the regions of interest. Amplified samples and assays were then loaded in to a 96.96 dynamic array (Fluidigm, USA) and the final detection qPCR was completed using the Biomark HD System (Fluidigm). Assays to detect CNAs were completed in quadruplicate and assays to detect fusion or mutations (Tables S5 and S6) were completed in duplicate. Any key drivers where a custom qPCR assay with the required sensitivity and specificity could not be designed were detected using single-cell Sanger sequencing (primer details in Table S7). The gene fusion, CNA, SNV and indel status for each single-cell was determined as previously described and the clonal phylogeny for the single-cell data was inferred using the maximum parsimony approach with PAUP* 4.0 for Linux.^4^ Inferred trees were visualized with the R package ape 4.0.^5^ Node support was assessed both with 100 nonparametric bootstrap replicates.

***Xenograft material***

Xenograft material was performed on stored samples (material from one xenograft per diagnostic case interrogated using single cell methods) and no statistical or blinding methods were used in this analysis.

***Cell lines***

In house stocks of cell line RPMI 8402 were authenticated by confirming the presence of the *STIL-TAL1* fusion and the sequence of previously published *PTEN* and *NOTCH1* mutations present in this cell line.

**Table 1. The genomic landscape of *STIL-TAL1* T-ALL.**

The following recurrent CNA T-ALL abnormalities were not detected in this subgroup: deletion 9q34.1 leading to *NUP214-ABL1* fusion; deletion *NF1* 17q11.2, deletion *PTPN2* 18p11.21 and deletion *PHF6* Xq26.2. Mutations in *IL7R* exon 6 were also screened for but not detected (in keeping with published data that IL7R exon 6 mutations are not detected in the *TAL/LMO* subgroup in T-ALL).

**REFERENCES**

1. Li H, Durbin R. Fast and accurate short read alignment with Burrows-Wheeler transform. *Bioinformatics* 2009 Jul 15; **25**(14)**:** 1754-1760.

2. McKenna A, Hanna M, Banks E, Sivachenko A, Cibulskis K, Kernytsky A*, et al.* The Genome Analysis Toolkit: a MapReduce framework for analyzing next-generation DNA sequencing data. *Genome Res* 2010 Sep; **20**(9)**:** 1297-1303.

3. McLaren W, Pritchard B, Rios D, Chen Y, Flicek P, Cunningham F. Deriving the consequences of genomic variants with the Ensembl API and SNP Effect Predictor. *Bioinformatics* 2010 Aug 15; **26**(16)**:** 2069-2070.

4. Wilgenbusch JC, Swofford D. Inferring evolutionary trees with PAUP*. *Current protocols in bioinformatics / editoral board, Andreas D Baxevanis [et al]* 2003 Feb; **Chapter 6:** Unit 6 4.

5. Paradis E, Claude J, Strimmer K. APE: Analyses of Phylogenetics and Evolution in R language. *Bioinformatics* 2004 Jan 22; **20**(2)**:** 289-290.

The raw data for the SNP6.0 arrays (CEL files) and whole exome sequencing (BAM files) experiments are all available on request. Microarray files are uploaded to ArrayExpress database, accession E-MTAB-6325.

**Supplementary tables**

**Table S1. Custom PCR primers used in Sanger sequencing**

| **Case and Driver Gene** | **Forward Primer** | **Reverse Primer** |
| --- | --- | --- |
| 6030 *FREM2* | CCCCATCACCTTAGTGCCTG | CCACTCTGTCACTGTTCGCT |
| 6030 *PTEN* ex 8 | AATCATGTGAATGAAAATGCAACAG | AGGTTTCCTCTGGTCCTGGTATG |
| 6116 *FREM2* | GCCCCTTTCCTCCTTCACTC | AGCCGTCTCTGTCATCATGC |
| 6116 *PIK3CD* | CGCAAACCTGTGACCCTCTC | GGCTTTCATGTGATCCCCCT |
| CF10 *PTEN* ex 5 | AATGGGGGAAAATAATACCTGGCT | AATCTAGGGCCTCTTGTGCC |

**Table S2. Details of FISH probes used in multicolour FISH**

| **Clone Name** | **Probe Name** | **Cytoband** | **CNA Loss or Gain** | **Relevant Driver Gene** | **False Positive Error Rate Normal Nuclei** |
| --- | --- | --- | --- | --- | --- |
| G248P80397F3  G248P82894B3  G248P8871D3  G248P81125E9 | STIL-TAL1 | 1p33 | Loss | *STIL-TAL1* Fusion | 2 |
| G248P89520G6 | CASP | 6q14.3 | Loss | *CASP8AP2* | 4 |
| CTD2104P21 | PTEN | 10q23.31 | Loss | *PTEN* | 2 |
| G248P82010F5 | p16.1 | 9p21.3 | Loss | *CDKN2A* | 4 |
| G248P82557D2 | p16.2 | 9p21.3 | Loss | *CDKN2B* | 3 |
| CTD3069A3 | MTAP | 9p21.3 | Loss | *MTAP* | 2 |
| RP11-11M9 | 4p | 4p15.31 | Loss | Unknown | 4 |

**Table S3. Mutation or fusion detection in single-cell qPCR. Primers used in amplification phase of single-cell experiments**

| **Case/ Driver Gene Target** | **Forward Primer** | **Reverse Primer** |
| --- | --- | --- |
| *STIL-TAL1* Fusion (All cases) | GGGGAGCTCGTGGGAGAAATTAAG | GCCTCGAAGGGTCCACATCTAC |
| 6030 *FREM2* | CCCCATCACCTTAGTGCCTG | CCACTCTGTCACTGTTCGCT |
| 6030 *PTEN* exon 8 | AATCATGTGAATGAAAATGCAACAG | AGGTTTCCTCTGGTCCTGGTATG |
| 6030 *PTEN* exon 7 | ATTTAACCATGCAGATCCTCAGTTT | GAACTCTACTTTGATATCACCACACACA |
| 6116 NOTCH1 | GTGCTGCACACCAACGTG | GAGGGCCCAGGAGAGTTG |
| 6116 *FREM2* | GCCCCTTTCCTCCTTCACTC | AGCCGTCTCTGTCATCATGC |
| 6116 *PIK3CD* | CGCAAACCTGTGACCCTCTC | GGCTTTCATGTGATCCCCCT |
| CF10 *PTEN* ex 5 | AATGGGGGAAAATAATACCTGGCT | AATCTAGGGCCTCTTGTGCC |
| CUL76 *NOTCH1* | AGACTGGCCCACCTCGTCTCT | AGGAACTGGGCTGCGGTC |
| CUL76 *SORC3* | CCATCTTCTTTCAGGACATGCA | TGGACCGCAGCAAATACTTG |

**Table S4. Copy number detection - commercial assays from Applied Biosystems and error rate assessed in cord blood cells (same assay used for amplification and detection steps)**

| **Gene or Region** | **Copy Number Assays** | **Error Rate (% loss or gain in diploid cells)** |
| --- | --- | --- |
| 1p33 (*STIL-TAL1*) | Hs00164774_cn; Hs02276370_cn; Hs02195778_cn | 6.2% |
| *CDKN2A* | Hs02738179_cn; Hs00237642_cn; Hs03699989_cn | 6.6% |
| 4p | Hs00968267_cn; Hs00446783_cn; Hs00791445_cn | 8.3% |
| 6q (*SYNCRIP*) | Hs02499447_cn; Hs02163835_cn; Hs01931036_cn | 5.2% |
| *LMO2* (upstream) | Hs05238266_cn; Hs05232616_cn; Hs05254034_cn | 4.2% |
| *PTEN* | Hs05214871_cn; Hs05096791_cn; Hs05152772_cn | 4.2% |
| *B2M* | Hs03896316_cn; Hs03900880_cn; Hs04450638_cn | NA (Control) |

**Table S5. SNV detection in single-cell qPCR: qPCR assays used in detection phase of single-cell experiments**

| **Case/ Driver Gene Target** | **Forward Primer** | **Reverse Primer** | **WT Probe (VIC)** | **MUT Probe (FAM)** | **False Positive (%)**  **Diploid Cells** |
| --- | --- | --- | --- | --- | --- |
| 6030 *FREM2* | GGATGACCAGCCACCTGTTC | TGTTTCACCCTCTGCCAGTGT | CAATGCCAACACG | CAATGCCAAAACGG | 0 |
| 6116 *FREM2* | GGTGAAGCCCATGAACACAATG | CCGAGACTGACCCTCATAGAGAATA | TCACCCGGAATACC | TCACCCAGAATACC | 0 |
| 6116 *PIK3CD* | AGCCGTTCCGCATCGA | AAACAGTGTCTCTCCCACCTATCC | CAAAGTGAACGCCGAC | CAGCAAAGTGAAAGC | 2 |
| CUL76 *SORC3* | CCATCTTCTTTCAGGACATGCA | TGGACCGCAGCAAATACTTG | ATCATCAGTACAGACGAG | ATCATCAGTACAGACTAG | 0 |

**Table S6. Fusion or indel detection in single-cell multiplex qPCR. qPCR assays used in detection phase of single-cell experiments**

| **Case/ Driver Gene Target** | **FAM labelled probe** | **Forward Primer Sequence** | **Reverse Primer Sequence** | **False positive (%) diploid cells** |
| --- | --- | --- | --- | --- |
| 6030 *PTEN* exon 7 WT | CACGACGGGAAGACA | GCCAGCTAAAGGTGAAGATATATTCCT | CGGCTGAGGGAACTCAAAGTA | 0 |
| 6030 *PTEN* exon 7 Indel 1 | TGGTCTGCCATTTAT | ATTTAACCATGCAGATCCTCAGTTT | CGTCGTGTGGGTCCTGGA | 0 |
| 6030 *PTEN* exon 7 Indel 2 | TTCCCTCAGATCCCTCCG | CCCACACGACGGGAAGAC | GAACTCTACTTTGATATCACCACACACA | 0 |
| 6030 *PTEN* exon 7 Indel 3 | CATGAACTTGTCTCGCC | TGCCAGCTAAAGGTGAAGATATATTC | ACGGCTGAGGGAACTCAAAG | 0 |
| B6116 *NOTCH1* | TCCCGAGTCCCC | CCAGCTACAGGTGCCTGAGC | CGGAGACGTTGGAATGCG | 0 |
| 6116 *STIL-TAL1* Fusion | TGGCTCGAGGTTTTAA | CGCTCTTGCATTCCTCACAA | AACCACGAAGAAGAAATGAAAACC | 0 |
| 6030 *STIL-TAL1* Fusion | CCCATAGAAAGTGTTTTC | TTCCTCACAATTTCTGGCTCATC | TGCACACAACCACGAAGAAGA | 0 |
| CUL76 *STIL-TAL1* Fusion | CACAATTTTTGTTGGTTTTC | TTGAATGCTCGCTCTTGCAT | GCACACAACCACGAAGAAGAAA | 2 |

**Table S7. Mutation detection in single-cell qPCR: primers used for single-cell Sanger sequencing**

| **Case/ Driver Gene Target** | **Forward Primer** | **Reverse Primer** |
| --- | --- | --- |
| CUL76 *NOTCH1* TAD domain | AGACTGGCCCACCTCGTCTCT | CTGAGCTCACGCCAAGGT |
| 6030 *PTEN* exon 8 | AATCATGTGAATGAAAATGCAACAG | AGGTTTCCTCTGGTCCTGGTATG |
| 6116 *FREM2* | GCCCCTTTCCTCCTTCACTC | AGCCGTCTCTGTCATCATGC |
| 6116 *PIK3CD* | CGCAAACCTGTGACCCTCTC | GGCTTTCATGTGATCCCCCT |
| 6116 *NOTCH1* | GTGCTGCACACCAACGTG | GAGGGCCCAGGAGAGTTG |

**Table S8. SNP 6.0 results. CNA detectable in samples interrogated by multicolour FISH. All measurements performed in CNAG using UCSC Genome Browser on Human Mar. 2006 (NCBI36/hg18) assembly**

| **Sample ID** | **Driver** | **Cytoband** | **Genomic Location** | **Copy Number Alteration** | **Genes (up to three listed)** |
| --- | --- | --- | --- | --- | --- |
| **6030** |  |  |  |  |  |
|  | *STIL-TAL1* | 1p33 | chr1:47,448,459-47,554,706 | loss | *STIL, TAL1* |
|  |  | 4p15.1 - 16.3 | chr4:26,568-30,928,582 | loss | *ZNF718, ZNF515, PIGG* |
|  | *6qdel* | 6q11.1 - 6q15 | chr6:62,384,976-88,923,095 | loss | *PTP4A1, PHF3, SYNCRIP* |
|  |  | 6q14.1 | chr6:79,019,345-79,098,352 | homologous loss | *Known CNV within above CNA* |
|  |  | 6q26 | chr6:162,380,474-162,584,354 | loss |  |
|  |  | 7p14.1 | chr7:38,269,645-38,323,600 | homologous loss | *TCRG, TARP* |
|  |  | 7q34 | chr7:141,671,521-142,046,676 | loss | *TCRB* |
|  |  | 7q34 | chr7:142,046,677-142,202,688 | homologous loss | *TCRB, PRSS1, PRSS2* |
|  | *CDKN2A, MTAP* | 9p21.3 | chr9:21,808,310-21,983,964 | homologous loss | *MTAP, CDKN2A* |
|  |  | 14q11.2 | chr14:21,646,254-22,012,459 | homologous loss | *TCRA, TCRD* |
|  |  | 19q13.42 | chr19:59,991,881-60,055,708 | loss | *KIR2DL4. KIR3DL1, KIR2DS4* |
|  |  | chromsome X | chrX | loss |  |
| **HK328** |  |  |  |  |  |
|  | *STIL-TAL1* | 1p33 | chr1:47,466,568-47,552,523 | loss | *STIL, TAL1* |
|  |  | 5p15.33-p12 | chr5:415,569-43,268,394 | gain | *CEP72, ADAMTS12, ZNF131* |
|  | *del 6q* | 6q13-q15 | chr6:71,922,222-92,907,817 | loss | *CASP8AP2, MAP3K7, SYNCRIP* |
|  |  | 7p14.1 | chr7:38,269,645-38,405,527 | homologous loss | *TCRG, TARP* |
|  |  | 7q34 | chr7:141,904,054-142,213,198 | homologous loss | *TCRB, PRSS1, PRSS2* |
|  |  | 9p21.3 | chr9:21,097,470-21,711,938 | loss | *IFNA21, IFNA16, IFNA7* |
|  | *CDKN2A/B* | 9p21.3 | chr9:21,711,939-22,334,340 | homologous loss | *CDKN2A, MTAP, CDKN2B* |
|  | *PAX5* | 9p13.2 - 21.3 | chr9:22,334,341-37,249,024 | loss | *ELAVL2, TEK, PAX5* |
|  |  | 14q11.2 | chr14:21,438,691-21,792,564 | loss | *TCRA, TCRD* |
|  |  | 14q11.2 | chr14:21,787,094-22,067,364 | homologous loss | *TCRA, TCRD* |
|  |  | chromsome X | chrX | loss |  |
| **Sample** | **Driver** | **Cytoband** | **Genomic Location** | **Copy Number Alteration** | **Genes (up to three listed)** |
| **CF6** |  |  |  |  |  |
|  | *STIL-TAL1* | 1p33 | chr1:47,480,956-47,564,950 | loss | *STIL,TAL1* |
|  |  | 7p14.1 | chr7:38,285,903-38,353,316 | homologous loss | *TCRG, TARP* |
|  |  | 7q34 | chr7:142,007,233-142,210,594 | homologous loss | *TCRB, PRSS1, PRSS2* |
|  |  | 9p21.3 - 24.3 | chr9:91,857-20,962,565 | loss | *SMARCA2, VLDLR, GLIS3* |
|  | *CDKN2A, CDKN2B, MTAP* | 9p21.3 | chr9:20,962,566-22,283,804 | homologous loss | *CDKN2A, MTAP, CDKN2B* |
|  |  | 9p21.2 - 21.3 | chr9:22,283805-26,279,217 | loss | *DMRTA1, ELAVL2, IZUMO3* |
|  |  | 9p21.2 | chr9:26,279,218-27,138,498 | gain | *CAAP1, PLAA, TEK* |
|  |  | 9p21.1-21.2 | chr9:27,138,499-31,765,814 | loss | *IFNK, LINGO2, EQTN* |
|  |  | 9p13.3 - 9p21.1 | chr9:31,765,815-32,957,267 | gain | *TAF1L, TMEM215, NDUFB6* |
|  |  | 9p13.3 - 9p21.1 | chr9:32,957,268-35,139,993 | loss | *SNORD121B, NUDT2, GALT* |
|  | *PAX5* | 9p13.1 - 9p13.3 | chr9:36,256,084-39,203,995 | loss | *PAX5, MELK, POLRIE* |
|  | *PTEN* | 10q23.31 | chr10:89,647,130-89,809,091 | loss | *PTEN* |
|  |  | 14q11.2 | chr14:21,516,441-22,001,467 | homologous loss | *TCRA, TCRD* |
|  |  | chromosome X | chrX | loss |  |
| **Sample** | **Driver** | **Cytoband** | **Genomic Location** | **Copy Number Alteration** | ***Genes (up to three listed)*** |
| **CF5** |  |  |  |  |  |
|  | *STIL-TAL1* | 1p33 | chr1:47,466,754-47,570,210 | loss | *STIL,TAL1* |
|  |  | 6q14.1 | chr6:77,479,216-77,517,792 | homologous loss |  |
|  |  | 7p14.1 | chr7:38,260,639-38,353,316 | homologous loss | *TCRG, TARP* |
|  |  | 7q34 | chr7:141,691,401-141,96987,073 | loss | *TCRB* |
|  |  | 7q34 | chr7:141,987,074-142,176,424 | homologous loss | *TCRB,PRSS1, PRSS2* |
|  | *CDKN2A, MTAP* | 9p21.3 | chr9:21,445,814-21,968,443 | homologous loss | *MTAP, CDKN2A* |
|  | *PTEN* | 10q23,1-10q23.31 | chr10:85,332,043-90,524,739 | loss | *RGR,BMPR1A, PTEN,* |
|  |  | 14q11.2 | chr14:21,378,629-21,811,946 | loss | *TCRA, TCRD* |
|  |  | 14q11.2 | chr14:21,811,947-22,040,167 | homologous loss | *TCRA, TCRD* |
|  |  | 15q15.3 | chr15:41,621,610-41,851,151 | loss | *PPIK5K1, CKMT1B, STRC* |

**Table S9. *NOTCH1* signalling pathway mutations in *STIL-TAL1* cohort**

| **Sample ID** | ***NOTCH1* Exons 26, 27 and 34 (PEST and Heterodimerisation domain)** | ***FBXW7* Exons 9 and 10** |
| --- | --- | --- |
| MH278 | WT | Ex9 c.1394G>A p.R465H |
| SC106 | WT | Ex9 c.1394G>A p.R465H |
| JA27 | WT | WT |
| HK328 | WT | WT |
| X562 | c.4867G>A p.E1623K | Ex9 c.1394G>A p.R465H |
| X2634 | WT SNP - c.5094C>T p.D1698D | WT |
| TP491 | c.4799T>A p.L1600Q; | WT |
| RPMI cell line | HD indel mutated; SNP - c.5094C>T p.D1698D | Ex9 c.1394G>A p.R465H |
| CF2 | WT | WT |
| CF5 | WT | WT |
| CF6 | c.7341C>T p.G2447G | WT |
| CF10 | WT SNP - c.5094C>T p.D1698D | WT |
| 6030 | WT | WT |
| B6116 | del c.7541_7542delCT p.P2514X | WT |
| CUL76 | c.7387_7388delG p.A2463PCPRRCHPRWSHPX; c.7021_7022insT p.S2341X | WT |
| 21922 | WT | WT |
| BR75 | WT SNP - c.5094C>T p.D1698D rs10521 | WT |
| BR74 | WT SNP c.5011G>A SNP - rs115563691; c.7515T>G SNP - rs34152221 | WT |
| S1 | c.4735_4737delGTG p.1578delV | WT |
| S2 | c.4735_4737delGTG p.1578delV | WT |

**Table S10. *PTEN* exon 7 mutations in *STIL-TAL1* cohort**

| **Sample ID** | ***PTEN* exon 7** |
| --- | --- |
| MH278 | WT |
| SC106 | WT |
| JA27 | c.695_696insGAGGGdelACGACGGGAAGAC p.T232TREVLVLX |
| HK328 | c.699_700insGACGGTGTdelCGGGAA p.R234DGVTSSCTLSSLSX |
| X562 | WT |
| X2634 | WT |
| TP491 | WT |
| RPMI Cell line | c.705_706insCCCCCGGCCC p.D236PPARQVHVLX |
| CF2 | WT |
| CF5 | > 1 sub-clonal mutation |
| CF6 | WT |
| CF10 | c.734_735del p.Q245X |
| 6030 | > 1 sub-clonal mutation |
| 6116 | WT |
| CUL76 | WT |
| 21922 | WT |
| BR75 | WT |
| BR74 | WT |
| S1 | WT |
| S2 | WT |

**Table S11 (a - d). Whole exome sequencing (WES) results, protein altering somatic SNVs present at read depth >20**

**Key for WES results tables**

- **Gene highlighted in blue = known or potential T-ALL driver***
- Highlighted yellow = verified by Sanger sequencing
- R = read depth diagnostic DNA
- M = mutated allele frequency
- Ref = reference allele and var = variant allele

*Mutations were designated potential driver status if the gene was known to be a key oncogene or tumour suppressor in leukaemia or if the mutation occurred in a key T-ALL signalling pathway or if the gene was listed in the Cosmic cancer gene census.

**Table S11a. 6030 protein altering somatic SNVs present at read depth >20**

| **Chr** | **Position** | **Ref** | **Var** | **Amino Acid** | [***Gene***](file:///C:\Users\caroline\Desktop\2017%20STIL_TAL1%20paper\1136-ICR_Report\html\varsBrowser\gene.htm%3ffile=AnnotatedVars\&geneid=ENSG00000197915) | **R** | **M** |
| --- | --- | --- | --- | --- | --- | --- | --- |
| **13** | **39263179** | **C** | **A** | **N566K** | [***FREM2***](file:///C:\Users\caroline\Desktop\2017%20STIL_TAL1%20paper\1136-ICR_Report\html\varsBrowser\gene.htm%3ffile=AnnotatedVars\&geneid=ENSG00000150893) | **50** | **20.8** |
| 1 | 152192314 | G | T | Y597X | [*HRNR*](file:///C:\Users\caroline\Desktop\2017%20STIL_TAL1%20paper\1136-ICR_Report\html\varsBrowser\gene.htm%3ffile=AnnotatedVars\&geneid=ENSG00000197915) | 32 | 37 |
| 14 | 24524467 | G | T | A185S | [*LRRC16B*](file:///C:\Users\caroline\Desktop\2017%20STIL_TAL1%20paper\1136-ICR_Report\html\varsBrowser\gene.htm%3ffile=AnnotatedVars\&geneid=ENSG00000186648) | 86 | 30.6 |
| 15 | 24923434 | T | A | V807E | [*NPAP1*](file:///C:\Users\caroline\Desktop\2017%20STIL_TAL1%20paper\1136-ICR_Report\html\varsBrowser\gene.htm%3ffile=AnnotatedVars\&geneid=ENSG00000185823) | 155 | 39.3 |
| 3 | 146312861 | T | C | I55V | [*PLSCR5*](file:///C:\Users\caroline\Desktop\2017%20STIL_TAL1%20paper\1136-ICR_Report\html\varsBrowser\gene.htm%3ffile=AnnotatedVars\&geneid=ENSG00000231213) | 26 | 48 |
| 3 | 38904699 | A | T | I1348N | [*SCN11A*](file:///C:\Users\caroline\Desktop\2017%20STIL_TAL1%20paper\1136-ICR_Report\html\varsBrowser\gene.htm%3ffile=AnnotatedVars\&geneid=ENSG00000168356) | 53 | 32.7 |
| 18 | 33706966 | G | A | A2V | [*SLC39A6*](file:///C:\Users\caroline\Desktop\2017%20STIL_TAL1%20paper\1136-ICR_Report\html\varsBrowser\gene.htm%3ffile=AnnotatedVars\&geneid=ENSG00000141424) | 36 | 20 |
| 2 | 31598283 | G | T | T522K | [*XDH*](file:///C:\Users\caroline\Desktop\2017%20STIL_TAL1%20paper\1136-ICR_Report\html\varsBrowser\gene.htm%3ffile=AnnotatedVars\&geneid=ENSG00000158125) | 74 | 37.5 |
| 19 | 58565078 | G | A | D296N | *ZCAN1* | 208 | 44.5 |

**Table S11b. 6116 protein altering somatic SNVs present at read depth >20**

| **Chr** | **Position** | **Ref** | **Var** | **Amino Acid** | **Gene** | **R** | **M** |
| --- | --- | --- | --- | --- | --- | --- | --- |
| 9 | 33385784 | C | G | E202D | [*AQP7*](file:///C:\Users\caroline\Desktop\2017%20STIL_TAL1%20paper\1136-ICR_Report\html\varsBrowser\gene.htm) | 24 | 21.7 |
| 22 | 17072482 | C | T | W320X | [*CCT8L2*](file:///C:\Users\caroline\Desktop\2017%20STIL_TAL1%20paper\1136-ICR_Report\html\varsBrowser\gene.htm%3ffile=AnnotatedVars\&geneid=ENSG00000198445) | 174 | 31 |
| **13** | **39262809** | **G** | **A** | **R443Q** | [***FREM2***](file:///C:\Users\caroline\Desktop\2017%20STIL_TAL1%20paper\1136-ICR_Report\html\varsBrowser\gene.htm%3ffile=AnnotatedVars\&geneid=ENSG00000150893) | **119** | **29.3** |
| MT | MT_11609 | T | C | S284P | [*MT-ND4*](file:///C:\Users\caroline\Desktop\2017%20STIL_TAL1%20paper\1136-ICR_Report\html\varsBrowser\gene.htm%3ffile=AnnotatedVars\&geneid=ENSG00000198886) | 26 | 34.6 |
| 20 | 30408121 | G | A | R82K | [*MYLK2*](file:///C:\Users\caroline\Desktop\2017%20STIL_TAL1%20paper\1136-ICR_Report\html\varsBrowser\gene.htm%3ffile=AnnotatedVars\&geneid=ENSG00000101306) | 94 | 38.4 |
| **1** | **9777666** | **C** | **A** | **N334K** | [***PIK3CD***](file:///C:\Users\caroline\Desktop\2017%20STIL_TAL1%20paper\1136-ICR_Report\html\varsBrowser\gene.htm%3ffile=AnnotatedVars\&geneid=ENSG00000171608) | **87** | **22.1** |
| 3 | 40503682 | C | A | Q203K | [*RPL14*](file:///C:\Users\caroline\Desktop\2017%20STIL_TAL1%20paper\1136-ICR_Report\html\varsBrowser\gene.htm%3ffile=AnnotatedVars\&geneid=ENSG00000188846) | 141 | 28.1 |
| 6 | 2766488 | G | C | R211P | [*WRNIP1*](file:///C:\Users\caroline\Desktop\2017%20STIL_TAL1%20paper\1136-ICR_Report\html\varsBrowser\gene.htm) | 23 | 21 |

**Table S11c. CF10 protein altering somatic SNVs present at read depth >20**

| **Chr** | **Position** | **Ref** | **Var** | **Amino Acid** | **Gene** | **R** | **M** |
| --- | --- | --- | --- | --- | --- | --- | --- |
|  |  |  |  |  |  |  |  |
| 22 | 43524615 | C | T | P125L | [*BIK*](file:///C:\Users\caroline\Desktop\2017%20STIL_TAL1%20paper\1136-ICR_Report\html\varsBrowser\gene.htm%3ffile=AnnotatedVars\&geneid=ENSG00000100290) | 73 | 37.5 |
| **10** | **88683389** | **G** | **A** | **W504X** | [***BMPR1A***](file:///C:\Users\caroline\Desktop\2017%20STIL_TAL1%20paper\1136-ICR_Report\html\varsBrowser\gene.htm%3ffile=AnnotatedVars\&geneid=ENSG00000107779) | **71** | **46.4** |
| 5 | 19520845 | A | T | V478D | [*CDH18*](file:///C:\Users\caroline\Desktop\2017%20STIL_TAL1%20paper\1136-ICR_Report\html\varsBrowser\gene.htm%3ffile=AnnotatedVars\&geneid=ENSG00000145526) | 137 | 50.4 |
| 11 | 71249492 | T | C | S131P | [*KRTAP5-8*](file:///C:\Users\caroline\Desktop\2017%20STIL_TAL1%20paper\1136-ICR_Report\html\varsBrowser\gene.htm%3ffile=AnnotatedVars\&geneid=ENSG00000241233) | 53 | 39.2 |
| 11 | 68171075 | G | A | R570Q | [*LRP5*](file:///C:\Users\caroline\Desktop\2017%20STIL_TAL1%20paper\1136-ICR_Report\html\varsBrowser\gene.htm%3ffile=AnnotatedVars\&geneid=ENSG00000162337) | 74 | 29.7 |
| 6 | 74191764 | G | T | G446V | [*MTO1*](file:///C:\Users\caroline\Desktop\2017%20STIL_TAL1%20paper\1136-ICR_Report\html\varsBrowser\gene.htm%3ffile=AnnotatedVars\&geneid=ENSG00000135297) | 88 | 36.8 |
| 10 | 55566598 | G | A | S1595L | [*PCDH15*](file:///C:\Users\caroline\Desktop\2017%20STIL_TAL1%20paper\1136-ICR_Report\html\varsBrowser\gene.htm%3ffile=AnnotatedVars\&geneid=ENSG00000150275) | 141 | 42.3 |
| 7 | 103197523 | A | T | F1900I | [*RELN*](file:///C:\Users\caroline\Desktop\2017%20STIL_TAL1%20paper\1136-ICR_Report\html\varsBrowser\gene.htm%3ffile=AnnotatedVars\&geneid=ENSG00000189056) | 147 | 44 |
| 11 | 61314676 | G | A | P107L | [*SYT7*](file:///C:\Users\caroline\Desktop\2017%20STIL_TAL1%20paper\1136-ICR_Report\html\varsBrowser\gene.htm%3ffile=AnnotatedVars\&geneid=ENSG00000011347) | 22 | 30 |
| 1 | 145439809 | G | C | G119R | [*TXNIP*](file:///C:\Users\caroline\Desktop\2017%20STIL_TAL1%20paper\1136-ICR_Report\html\varsBrowser\gene.htm%3ffile=AnnotatedVars\&geneid=ENSG00000117289) | 165 | 31.9 |

**Table S11d. CF5 protein altering somatic SNVs present at read depth >20**

| **Chr** | **Position** | **Ref** | **Var** | **Amino Acid** | **Gene** | **R** | **M** |
| --- | --- | --- | --- | --- | --- | --- | --- |
| 7 | 48494732 | G | A | A4222T | [*ABCA13*](file:///C:\Users\caroline\Desktop\2017%20STIL_TAL1%20paper\1136-ICR_Report\html\varsBrowser\gene.htm%3ffile=AnnotatedVars\&geneid=ENSG00000179869) | 91 | 51.1 |
| 10 | 101147624 | C | A | D796E | [*CNNM1*](file:///C:\Users\caroline\Desktop\2017%20STIL_TAL1%20paper\1136-ICR_Report\html\varsBrowser\gene.htm%3ffile=AnnotatedVars\&geneid=ENSG00000119946) | 160 | 44.5 |
| 4 | 187455677 | G | C | D73E | [*MTNR1A*](file:///C:\Users\caroline\Desktop\2017%20STIL_TAL1%20paper\1136-ICR_Report\html\varsBrowser\gene.htm%3ffile=AnnotatedVars\&geneid=ENSG00000168412) | 157 | 42.5 |
| 17 | 16068377 | C | G | K178N | [*NCOR1*](file:///C:\Users\caroline\Desktop\2017%20STIL_TAL1%20paper\1136-ICR_Report\html\varsBrowser\gene.htm%3ffile=AnnotatedVars\&geneid=ENSG00000141027) | 24 | 41.7 |
| X | 118723457 | C | A | G644V | [*NKRF*](file:///C:\Users\caroline\Desktop\2017%20STIL_TAL1%20paper\1136-ICR_Report\html\varsBrowser\gene.htm%3ffile=AnnotatedVars\&geneid=ENSG00000186416) | 141 | 47.2 |
| 2 | 138400118 | C | T | P1257L | [*THSD7B*](file:///C:\Users\caroline\Desktop\2017%20STIL_TAL1%20paper\1136-ICR_Report\html\varsBrowser\gene.htm%3ffile=AnnotatedVars\&geneid=ENSG00000144229) | 101 | 52.5 |
| 8 | 77776539 | T | C | L3530P | [*ZFHX4*](file:///C:\Users\caroline\Desktop\2017%20STIL_TAL1%20paper\1136-ICR_Report\html\varsBrowser\gene.htm%3ffile=AnnotatedVars\&geneid=ENSG00000091656) | 222 | 50.5 |

**Table S12 (a – d). WES results, protein altering indels present at read depth >20**

**Table S12a. 6030 protein altering indels present at read depth >20 ****

| **Chr** | **Position** | **Ref/Var** | **Amino Acid** | **Gene** | **R** | **M** |
| --- | --- | --- | --- | --- | --- | --- |
| 6 | 16327865 | -/TGC | H226indel | *ATXN1* | 57 | 32.1 |
| 1 | 240370914 | GCCCCCTCTACCCGGAGCGGGAATACCTCCTC/- | L934indel | [*FMN2*](file:///C:\Users\caroline\1136-ICR_Report\html\varsBrowser\gene.htm%3ffile=AnnotatedVars\&geneid=ENSG00000155816) | 30 | 30 |
| 14 | 106329451 | -/ACC | M7indel | [*IGHJ6*](file:///C:\Users\caroline\1136-ICR_Report\html\varsBrowser\gene.htm%3ffile=AnnotatedVars\&geneid=ENSG00000211900) | 91 | 46 |
| **10** | **89720677** | **T/-** | N276indel | [***PTEN***](file:///C:\Users\caroline\1136-ICR_Report\html\varsBrowser\gene.htm%3ffile=AnnotatedVars\&geneid=ENSG00000171862) | **118** | **25.5** |

**Table S12b. 6116 protein altering indels present at read depth >20 ****

| **Chr** | **Position** | **Ref/Var** | **Amino Acid** | **Gene** | **R** | **M** |
| --- | --- | --- | --- | --- | --- | --- |
| 7 | 128587352 | TGCAGCCGCCCACTCTGCGGCCGCCT/- | P156indel | *IRF5* | 41 | 51 |
| 10 | 3208568 | -/GCACGCTAGGGAAGAGAGA | Q91indel | *PITRM* | 29 | 22 |
| 1 | 152084176 | -/TGCTGCTCGCGCCTCTCC | Q506indel | *TCHH* | 185 | 21 |

**Table S12c. CF10 protein altering indels present at read depth >20**

| **Chr** | **Position** | **Ref/Var** | **Amino Acid** | **Gene** | **R** | **M** |
| --- | --- | --- | --- | --- | --- | --- |
| 14 | 92537386 | TTT/- | K240indel | *ATXN3* | 35 | 25 |
| 15 | 90320121 | AGGGGCAGGGGCAAGGGCAGGGGC/ - | E178indel | *MESP2* | 31 | 67 |
| **10** | **89692811** | **-/A** | **E99indel** | ***PTEN*** | **203** | **25.3** |
| 7 | 44805118 | -/C | A670indel | *ZMIZ2* | 80 | 28.9 |

**Table S12d. CF5 protein altering indels present at read depth >20**

| **Chr** | **Position** | **Ref/Var** | **Amino Acid** | **Gene** | **R** | **M** |
| --- | --- | --- | --- | --- | --- | --- |
| 14 | 105350741 | -/C | T542indel | *CEP170B* | 33 | 26.7 |
| 14 | 106329451 | -/ACC | M1indel | [*IGHJ6*](file:///F:\1136-ICR_Report\html\varsBrowser\gene.htm%3ffile=AnnotatedVars\&geneid=ENSG00000211900) | 112 | 31.8 |
| 12 | 53207584 | -/CACCAAAGCCACCAGTGCCGAAAC | G87indel | [*KRT4*](file:///F:\1136-ICR_Report\html\varsBrowser\gene.htm%3ffile=AnnotatedVars\&geneid=ENSG00000170477) | 83 | 60.6 |
| **X** | **133511691** | **-/AAAAA** | R15indel | [***PHF6***](file:///F:\1136-ICR_Report\html\varsBrowser\gene.htm%3ffile=AnnotatedVars\&geneid=ENSG00000156531) | **173** | **34.7** |
| 6 | 170871014 | -/CAG | Q64indel | [*TBP*](file:///F:\1136-ICR_Report\html\varsBrowser\gene.htm%3ffile=AnnotatedVars\&geneid=ENSG00000112592) | 65 | 70.3 |

** The bio-informatics pipeline did not detect the known *NOTCH1* indel present in 6116 or the multiple low level *PTEN* exon 7 indels suggested by previous Sanger sequencing.

Table S13. Functional effects of *PTEN* indels

| **Sample and Mutation** | ***PTEN* exon** | **Indel Mutation Details** | **Functional Effect** |
| --- | --- | --- | --- |
| 6030 Indel 1 | 7 | c.657_685delGCTAAAGGTGAAGATATATTCCTCCAATTinsTTTATCGTC; p.Q219HLSSRTHTTGRQVHVLX | Stop Codon |
| 6030 Indel 2 | 7 | c.735_736insATCCCT; p.245_246insIP | In frame ins (2 AA insertion) |
| 6030 Indel 3 | 7 | c.699_704delACGGGAinsGGGGGGCG; p.R233RGGETSSCTLSSLSRYLCVVISKX | Stop Codon |
| 6030 Indel 4 | 7 | c.684_701delTTCAGGACCCACACGACGinsCCCAGGTGAC; p.N228NPGDGRQVHVLX | Stop Codon |
| 6030 Exon 8 Indel | 8 | c.828delT; p.N276KHSSYQDQRKPQKKX | Stop Codon |
| CF5 Indel A | 7 | c.694_700delACACGACinsTCAGCTTACCCCACTCCT; p.T232SAYPTPGKTSSCTLSSLSRYLCVVISKX | Stop Codon |
| CF5 Indel B | 7 | c.696_701delACGACGinsCCAGGGAGTA; p.T232TQGVGRQVHVLX | Stop Codon |
| CF5 Indel C | 7 | c.686_698delCAGGACCCACACG; p.S229YGKTSSCTLSSLSRYLCVVISKX | Stop Codon |
| CF5 Indel D * | 7 | c.696_697insCCGAATTC; p.R233PNSDGKTSSCTLSSLSRYLCVVISKX | Stop Codon |
| CF10 Exon 5 Indel | 5 | c.297_298insA; p.L100TYQTLLX | Stop Codon |
| CF10 Exon 7 Indel | 7 | c734_735del p.Q245X ** | Stop Codon |

Table based on data from Furness et al as well as previously published data by Jenkinson, Gale et al for sample CF5.

- *The presence of at least 4 low-level exon 7 indels in CF5 was suggested by heteroduplex analysis; 3 were validated by cloning. The sequence of the 4^th^ mutant presented is based on the size inferred by HPLC and a corresponding size indel noted in two reads in the next generation sequencing data. All other exon 7 indels in the table were resolved to single bp resolution by cloning
- **CF10 exon 7 indel resulted in a stop codon but the 3’ breakpoint of the indel was undetermined so this was not included in the MEME or RSS analysis.

**Table S14. RSS scores *PTEN* indels. Positive scores are highlighted grey and negative scores white**

| **Name** | **Start POS1** | **End POS1** | | **Start POS2** | **End POS2** | | **Score 1**  **POS 1** | **Score 2**  **POS 1** | | **Score 3**  **POS 1** | | **Score 4**  **POS 1** | | | **Score 1**  **POS 2** | **Score 2**  **POS 2** | | **Score 3**  **POS 2** | **Score 4**  **POS 2** | |  |
| --- | --- | --- | --- | --- | --- | --- | --- | --- | --- | --- | --- | --- | --- | --- | --- | --- | --- | --- | --- | --- | --- |
| CF10 Exon_5_Indel | 87933037 | 87933077 | | 87933038 | 87933078 | | 6.463058 | -1.316716 | | -1.6955924 | | 0.4753070 | | | 6.7529460 | -1.0268291 | | -1.9854799 | 0.1854194 | |  |
| CF5 Exon_7_Indel_A | 87957892 | 87957932 | | 87957900 | 87957940 | | -4.115255 | -6.4452551 | | 0.693620 | | -2.9449178 | | | -1.7961545 | -4.1261545 | | -1.6254799 | -0.3769415 | |  |
| CF5 Exon_7_Indel_B | 87957894 | 87957934 | | 87957901 | 87957941 | | -3.53547 | -5.8654799 | | 0.1138454 | | -3.5246929 | | | -1.5062661 | -3.8362669 | | -1.915367 | -0.6668291 | |  |
| CF5 Exon_7_Indel_C | 87957884 | 87957924 | | 87957898 | 87957938 | | -2.97682 | -8.7643556 | | 3.0127211 | | -0.6258172 | | | -2.3759297 | -4.7059297 | | -1.0457048 | 0.2028335 | |  |
| CF5 Exon_7_Indel_D | 87957895 | 87957935 | | 87957896 | 87957936 | | -3.24559 | -5.5755924 | | -0.1760421 | | -3.8145805 | | | -2.9557048 | -5.2857048 | | -0.4659297 | -3.6457496 | |  |
| 6030 Exon_7_Indel_1 | 87957855 | 87957895 | | 87957894 | 87957934 | | 3.35272 | 2.7029460 | | -1.5834562 | | 6.1865437 | | | -3.5354799 | -5.8654799 | | 0.1138454 | -3.5246929 | |  |
| 6030 Exon_7_Indel_2 | 87957934 | 87957974 | | 87957935 | 87957975 | | -5.08716 | -1.8543556 | | -11.095592 | | -5.9255924 | | | -4.7972788 | -1.5644681 | | -11.385479 | -6.2154799 | |  |
| 603 Exon_7_Indel_3 | 87957897 | 87957937 | | 87957905 | 87957945 | | -2.66581 | -4.9958172 | | -0.7558172 | | 0.4927211 | | | -0.3467167 | -2.6767167 | | -3.0749178 | -1.8263794 | |  |
| 6030 Exon_7_Indel_4 | 87957882 | 87957922 | | 87957901 | 87957941 | | -3.55660 | -9.3441308 | | -2.3652551 | | -0.0460421 | | | -1.5062669 | -3.8362669 | | -1.915367 | -0.6668291 | |  |
| 6030 Exon_8_Indel | 87960900 | 87960940 | 87960902 | | 87960942 | -1.82671 | | | -4.045142 | | -7.6762669 | | -10.476604 | -1.2469415 | | | -3.465367 | 6.3627211 | | 6.3627211 | |

Score 1 = 12bp spacer 3' End; Score 2 = 23bp spacer 3' End; Score 3 = 12bp spacer 5' End; Score 4 = 23bp spacer 5' End

**Table S15. *STIL-TAL1* fusion sequences**

| **Sample ID** | ***STIL*-** | **N Nucleotides** | **-*TAL1*** |
| --- | --- | --- | --- |
| 6030 | CAATTTCTGGCTCA | TCTCCCATAGAAAGT | GTTTTCATTTCTTC |
| 6116 | CAATTTCTGGCTC | GAGGTTTTAAGGG | GGTTTTCATTTCTT |
| CF10 | GCATTCCTCACAAT | ACCTA | TTTTCATTTCTTCTT |
| JA27 | TCACAATTTCTGGC | CCTTACTG | TTGGTTTTCATTTCT |
| SC106 | CTCACAATTTCTGG | GTGCCTAG | GGTTTTCATTTCTTC |
| CUL76 | ATTCCTCACAATTT | TT | GTTGGTTTTCATTTC |

**Table S16a-c. Xenograft Multiplex qPCR analysis, sub-clones present in xenograft and relative proportions. Sub-clone labelling C1, C2 etc corresponds to sub-clones detected in diagnostic sample in Figure 2. S16a 6116 Xenograft Single Cell Data (N = 180 cells)**

| **Sub-Clone** | **Genetic Profile** | **%** |
| --- | --- | --- |
| C1 | *STIL-TAL1 F+*  *0 CDKN2A*  *FREM2+* | 4.4 |
| C2 | *STIL-TAL1 F+*  *0 CDKN2A*  *PIK3CD+* | 6.7 |
| C3 | *STIL-TAL1* F+  *0 CDKN2A*  *PIK3CD*+  *FREM2*+ | 66.7 |
| C4 | *STIL-TAL1* F+  *CDKN2A* 0 copies  *PIK3CD*+  *FREM2*+  *NOTCH1*+ | 22.2 |

S16b CUL76 Xenograft Single Cell Data (N = 145 cells)

| **Sub-Clone** | **Genetic Profile** | **%** |
| --- | --- | --- |
| C1 | *STIL-TAL1 F+*  *0 CDKN2A*  *1 LMO2*  *1 PTEN* | 87.6 |
| C2 | *STIL-TAL1 F+*  *0 CDKN2A*  *1 LMO2*  *1 PTEN*  *SORC3+* | 7.6 |
| C3 | *STIL-TAL1 F+*  *0 CDKN2A*  *1 LMO2* | 4.8 |

S16c 6030 Xenograft Single Cell Data (N = 240 cells)

| **Sub-Clone** | **Genetic Profile** | **%** |
| --- | --- | --- |
| C1 | *STIL-TAL1 F+*  *0 CDKN2A* | 100** |

**Sanger sequencing on 78 cells performed to ascertain *PTEN* exon 8 mutation status, 69 showed homozygous deletion (88%) and 9 (12%) showed heterozygous deletion of *PTEN* exon 8

**Supplementary figure legends**

**Supplementary Figure S1a. Evolutionary trees based on multicolour FISH data for sample 6030 and Xenograft.** Abnormal FISH signal patterns seen in diagnostic material from sample 6030. Each genetically distinct abnormal sub-clone is characterised by a unique FISH signal pattern. The earliest detectable abnormal sub-clone has the *STIL-TAL1* fusion and one copy of the region 9p21.3. FISH probes STIL-TAL1 (spectrum red-green), p16.1 (spectrum Cy3, coloured orange in photographs), SYNC (6q, spectrum aqua) and 4p (biotin-Cy5, coloured pink in photographs) were used in this experiment. Copy number losses of 4p and 6q were sub-clonal events. The data shown is based on the use of FISH probe p16.1 but the experiment was repeated using a larger 9p21.3 FISH probe for the *MTAP* region (to verify the presence of the 9p21.3 region copy number 1 clone) with a similar frequency of the purple sub-clone with one copy of 9p21.3 detected.

Limited xenograft material was available for FISH (40 nuclei assessed). Four rather than five colour FISH was performed due to sample quality and only FISH probes >80kbp in size were used (using probes STIL-TAL1, spectrum red-green); MTAP (9p21.3, Cy3 coloured blue); and 6q (biotin-Cy5 coloured pink). Assessment of 4p copy number status was ascertained separately (6q, biotin-Cy5 coloured yellow) and 4p (Cy3 coloured pink). Combined results of two experiments confirmed that only cells derived from the purple sub-clone were present in the xenograft transplant, i.e. *STIL-TAL1* fusion positive, 6q two copies and 4p two copies and 9p one copy.

**Supplementary Figure S1b. Review of FISH *versus* multiplex qPCR data in 6030 and 6030 xenograft implies re-iterative *CDKN2A* deletion.** Bulk SNP array analysis in 6030 diagnostic and xenograft DNA is shown here. The dominant 9p21.3 deletion in the patient showed loss of two copies in both area A, which includes the MTAP FISH probe, and Area B. The dominant 9p21.3 deletion in the xenograft showed mono-allelic loss in Area A but bi-allelic loss in area B. Even a small in-house fosmid 40kb FISH probe was larger than the bi-allelically deleted region in the xenograft. Single-cell multiplex qPCR assays used sit within the *CDKN2A* gene only, i.e. within area B only, explaining the discrepant FISH and multiplex qPCR results. This data implies that both phylogenetic trees generated by FISH and qPCR are an oversimplification and that 9p deletion is therefore likely to be a re-iterative and secondary event occurring sub-clonally to the *STIL-TAL1* fusion as in both 6030 and the xenograft material all abnormal clones have bi-allelic *CDKN2A* deletions but the breakpoint of the deletion on at least one allele is distinct between diagnostic and xenograft material.

**Supplementary Figure S2. Validation of re-iterative subclone in 6116.** Sanger sequencing of single-cells in the 6116/6116 xenograft to validate qPCR data showing that both *PIK3CD* mutated *FREM2* wild-type (WT) and *FREM2* WT *PIK3CD* mutated cells were present. One of these mutations had therefore occurred re-iteratively.

**Supplementary Figure S3. Localisation of *FREM2* mutations.** Mutation localisation within FREM2 protein. FREM2 protein structure with localisation of mutations for 6030 (p.N566K) and 6116 (p.R443Q) (GRCh37/hg19 using NCBI references NM_207361 and NP_997244). Both are missense mutations but outside Calx-beta domains.
