# Supplementary figures and images for: The subclonal complexity of STIL-TAL1+ T-cell acute lymphoblastic leukaemia
Source: Leukemia. 2018 Mar 20;32(9):1984–93. doi: 10.1038/s41375-018-0046-8 (PMC6127084; doi:10.1038/s41375-018-0046-8)

## Slide 1
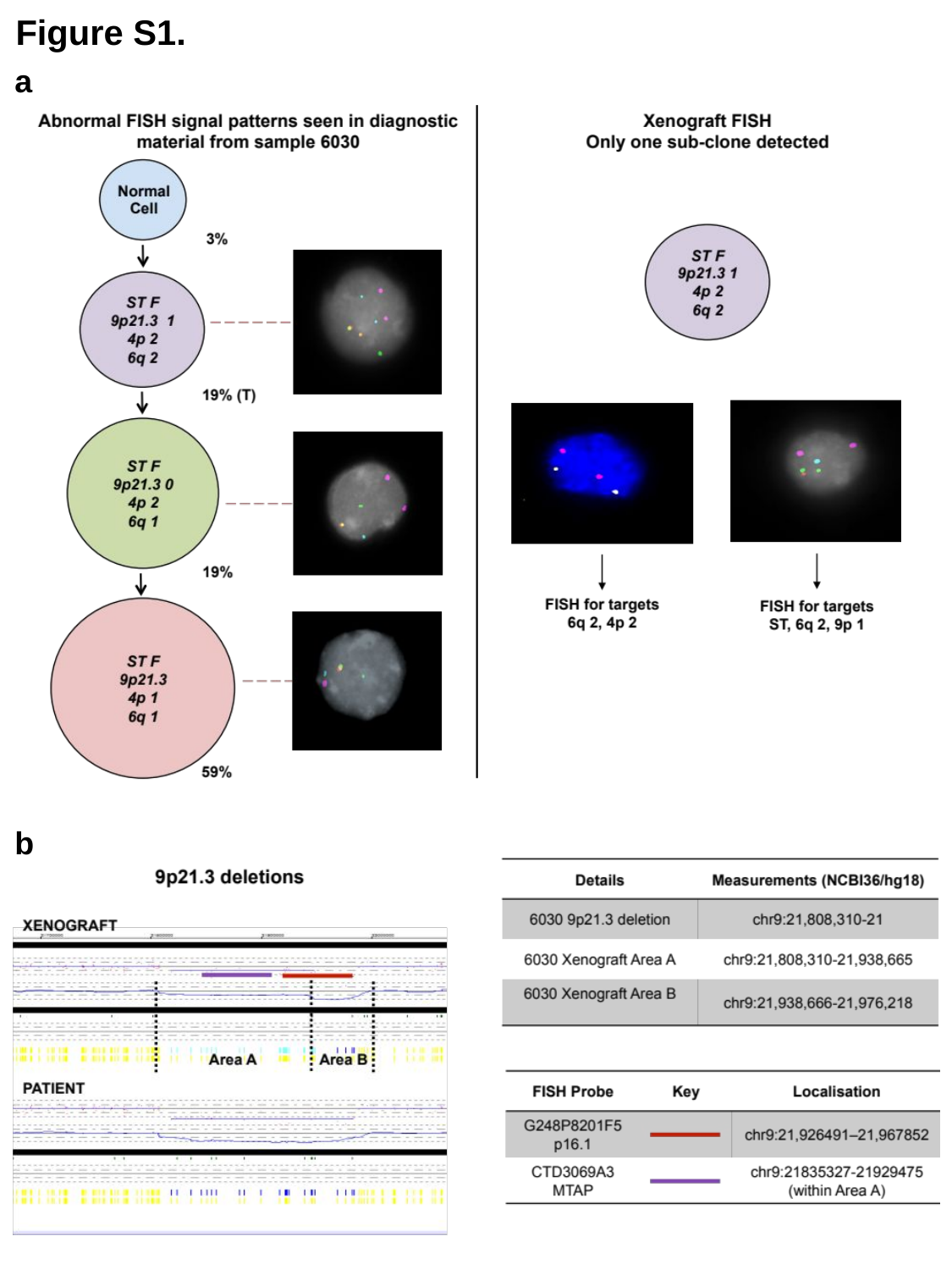

Figure S1.
a
b

Supplement: Supplementary file 2 — Figure S1(PPTX 691 kb) [file 41375_2018_46_MOESM2_ESM.pptx]

## Slide 1
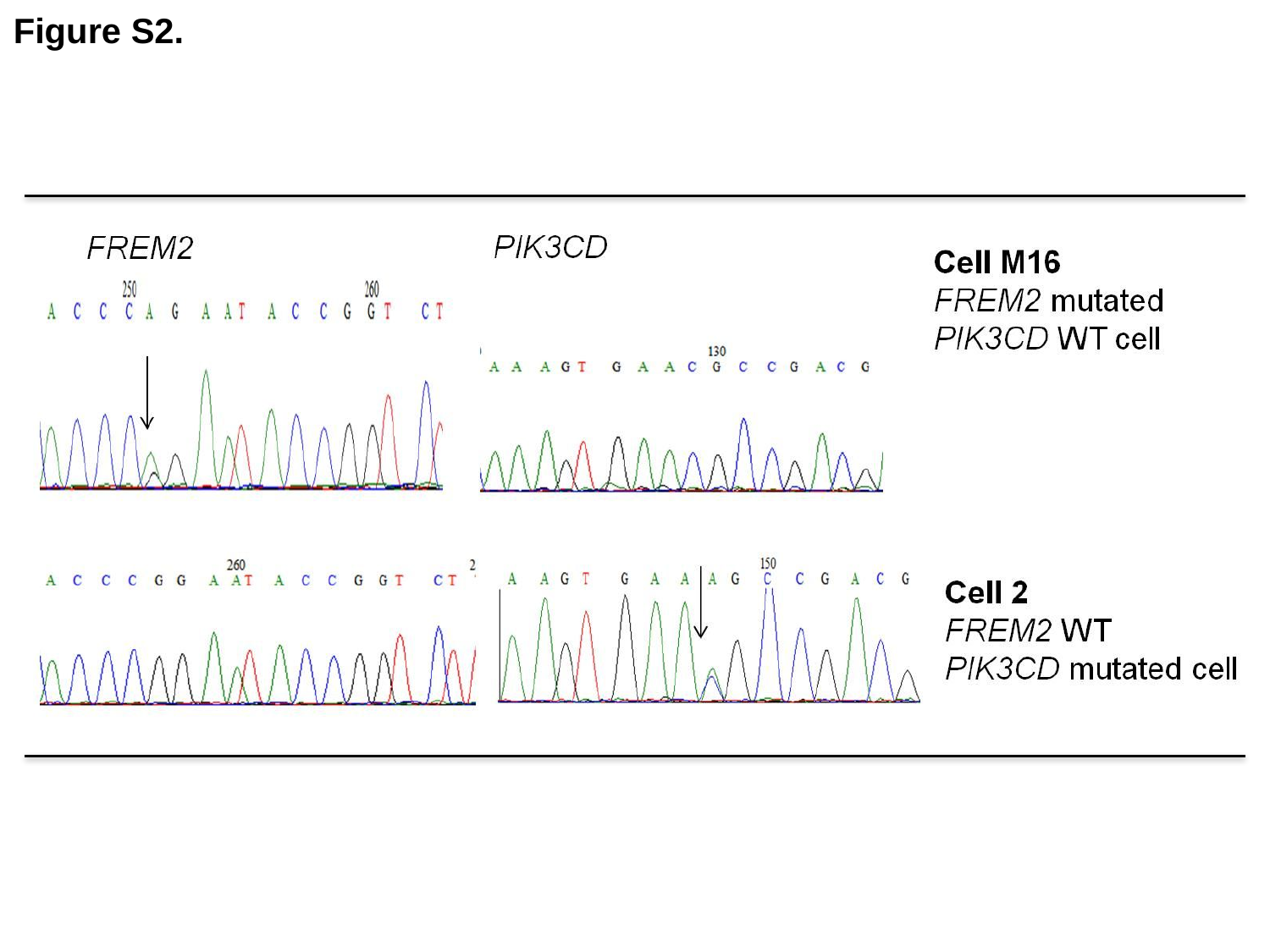

Figure S2.
#

Supplement: Supplementary file 3 — Figure S2a,b(PPTX 154 kb) [file 41375_2018_46_MOESM3_ESM.pptx]

## Slide 1
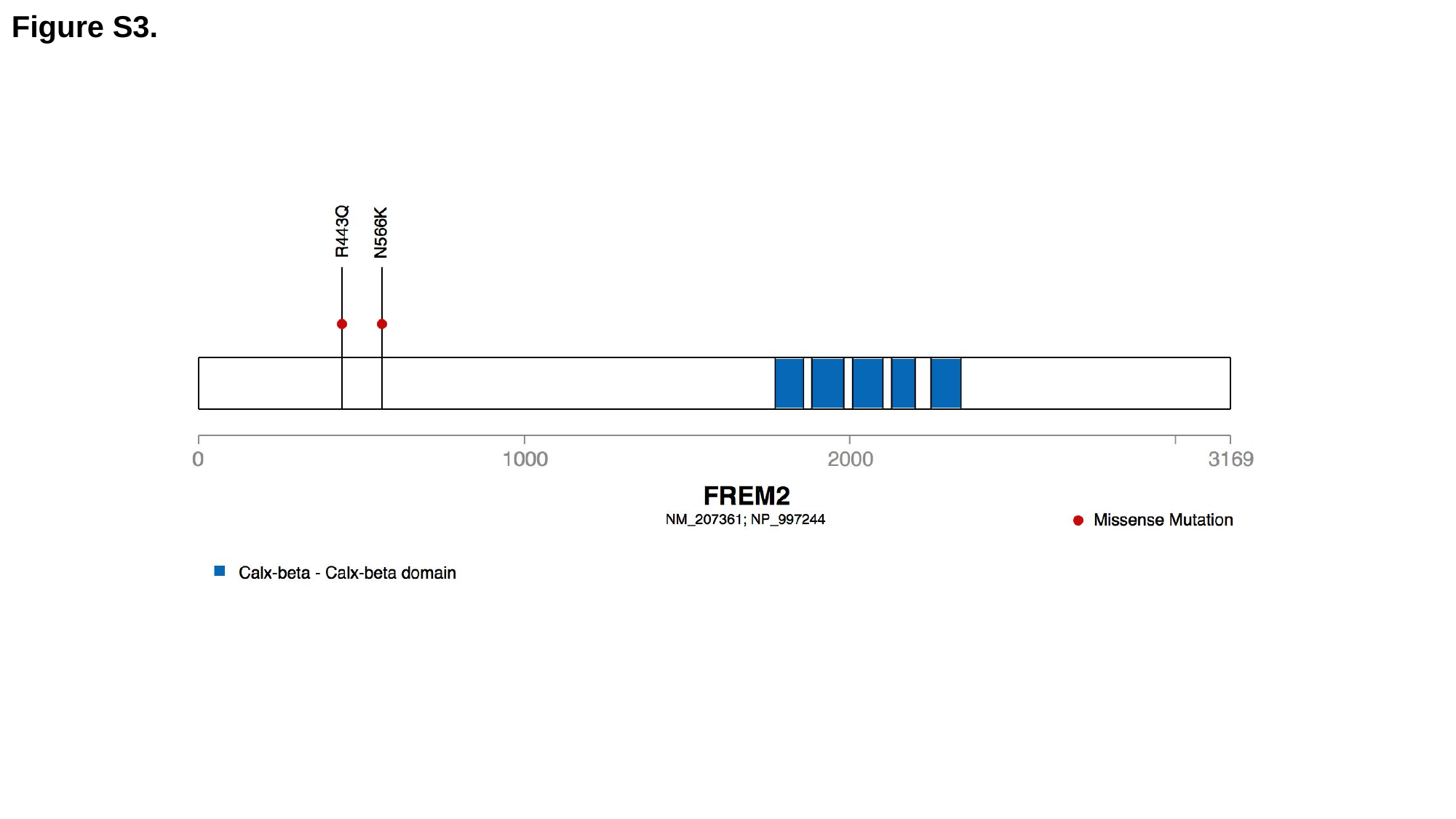

Figure S3.

Supplement: Supplementary file 4 — Figure S3(PPTX 92 kb) [file 41375_2018_46_MOESM4_ESM.pptx]
